# Supplementary material for: Comparison of quantitative ultrasonography and dual X-ray absorptiometry for bone status assessment in South African children living with HIV
Source: PLoS One. 2022 Oct 17;17(10):e0276290. doi: 10.1371/journal.pone.0276290 (PMC9576091; doi:10.1371/journal.pone.0276290)
Supplement: S1 File — (DOCX) [file pone.0276290.s001.docx]

**SUPPLEMENTARY MATERIAL**

**S1 Table. Baseline comparisons between participants with calcaneal QUS scans and those missing calcaneal QUS scans at baseline or 12-months.**

|  | With QUS (n = 279) | Without QUS (n = 144) | p-value |
| --- | --- | --- | --- |
| Age, Mean (SD) | 6.74 (1.43) | 6.54 (1.41) | 0.169 |
| Sex, n (%)  Male  Female | 144 (51.6%)  135 (48.4%) | 81 (56.3%)  63 (43.7%) | 0.205 |
| Weight (kg)  Mean (SD) | 20.80 (4.80) | 20.92 (5.36) | 0.824 |
| Weight-for-age *z*-score  Mean (SD) | -0.60 (1.00) | -0.46 (1.08) | 0.220 |
| Underweight, n (%)  No  Yes | 256 (91.8%)  23 (8.2%) | 136 (94.4%)  8 (5.6%) | 0.734 |
| Height (cm)  Mean (SD) | 113.71 (9.64) | 113.2 (9.23) | 0.588 |
| Height-for-age  *z*-score  Mean (SD) | -1.14 (0.96) | -1.04 (0.94) | 0.328 |
| Stunted, n (%)  No  Yes | 218 78.1%)  61 (21.9%) | 124 (86.1%)  20 (13.9%) | 0.134 |
| BMI-for-age *z*-score  Mean (SD) | 0.16 (1.00) | 0.25 (1.10) | 0.384 |
| Tanner Stage, n (%)  Tanner 1  Tanner 2 | 275 (98.6%)  4 (1.4%) | 143 (99.3%)  1 (0.7%) | 1.00 |
| CLHIV, n (%) | 166 (59.5%) | 52 (36.1%) | <0.001 |
| CD4+ count (cells/μl), mean (SD) | 1209.21 (417.69) | 1227.49 (443.90) | 0.795 |

**S2 Table. Baseline comparisons between participants with normal calcaneal SOS values and those excluded for abnormally elevated calcaneal SOS (>1625 m/s).**

|  | Normal Calcaneal SOS (n = 170) | Abnormal Calcaneal SOS (n = 109) | p-value |
| --- | --- | --- | --- |
| Age, Mean (SD) | 7.21 (1.46) | 5.78 (0.69) | <0.001 |
| Sex, n (%)  Male  Female | 91 (53.5%)  79 (46.5%) | 53 (48.6%)  56 (51.4%) | 0.581 |
| Weight (kg)  Mean (SD) | 22.70 (5.06) | 17.30 (2.00) | <0.001 |
| Weight-for-age *z*-score  Mean (SD) | -0.33 (1.09) | -1.06 (0.82) | <0.001 |
| Underweight, n (%)  No  Yes | 161 (94.7%)  9 (5.3%) | 95 (87.2%)  14 (12.8%) | 0.040 |
| Height (cm)  Mean (SD) | 117.13 (8.65) | 106.20 (5.19) | <0.001 |
| Height-for-age  *z*-score  Mean (SD) | -0.95 (0.89) | -1.60 (0.83) | <0.001 |
| Stunted, n (%)  No  Yes | 145 (85.3%)  25 (14.7%) | 73 (67.0%)  36 (33.0%) | <0.001 |
| BMI-for-age *z*-score  Mean (SD) | 0.33 (1.13) | -0.04 (0.90) | 0.002 |
| Tanner Stage, n (%)  Tanner 1  Tanner 2 | 167 (98.2%)  3 (1.8%) | 108 (99.1%)  1 (0.09%) | 0.278 |
| CLHIV, n (%) | 80 (47.1%) | 86 (78.9%) | <0.001 |
| CD4+ count (cells/μl), mean (SD) | 1185.50 (372.98) | 1220.69 (428.17) | 0.572 |

**S3 Table. Baseline comparisons between participants with radial (Mini Omnisense) QUS scans and those missing radial QUS scans at baseline or 12-months.**

|  | With QUS (n = 205) | Without QUS (n = 218) | p-value |
| --- | --- | --- | --- |
| Age, Mean (SD) | 6.76 (1.40) | 6.60 (1.45) | 0.251 |
| Sex, n (%)  Male  Female | 105 (51.2%)  100 (48.8%) | 114 (52.3%)  104 (47.7%) | 0.902 |
| Weight (kg)  Mean (SD) | 20.88 (4.91) | 20.79 (5.06) | 0.856 |
| Weight-for-age *z*-score  Mean (SD) | -0.59 (0.99) | -0.52 (1.07) | 0.524 |
| Underweight, n (%)  No  Yes | 194 (94.6%)  9 (4.4%) | 200 (91.7%)  18 (8.3% | 0.325 |
| Height (cm)  Mean (SD) | 114.48 (9.50) | 112.65 (9.45) | 0.048 |
| Height-for-age  *z*-score  Mean (SD) | -1.01 (0.98) | -1.20 (0.92) | 0.043 |
| Stunted, n (%)  No  Yes | 172 (83.9%)  33 (16.1%) | 173 (79.4%)  45 (20.6%) | 0.280 |
| BMI-for-age *z*-score  Mean (SD) | 0.05 (1.01) | 0.32 (1.05) | 0.008 |
| Tanner Stage, n (%)  Tanner 1  Tanner 2 | 202 (98.5%)  3 (1.5%) | 216 (99.1%)  2 (0.9%) | 0.945 |
| CLHIV, n (%) | 101 (49.3%) | 117 (53.7%) | 0.419 |
| CD4+ count (cells/μl), mean (SD) | 1281.22 (459.19) | 1155.02 (381.43) | 0.030 |

**S4 Table. Comparison of absolute change between baseline and 12-month follow-up in QUS and DXA measures between CLHIV and uninfected controls.**

|  | **Calcaneal QUS** | | | **Radial QUS** | | |
| --- | --- | --- | --- | --- | --- | --- |
|  | CLHIV (n=80) | HIV- Control (n=90) | p-value | CLHIV (n=101) | HIV- Control (n=104) | p-value |
| SOS, mean (SD) | 8.213 (21.3) | 1.599 (20.4) | 0.041 | 14.61 (105) | -13.49 (122) | 0.0808 |
| BUA, mean (SD) | 3.633 (15.2) | 9.124 (13.3) | 0.014 | NA | NA | NA |
| Stiffness Index, mean (SD) | 4.825 (12.4) | 6.629 (10.8) | 0.316 | NA | NA | NA |
| Whole Body BMC, mean (SD) | 98.87 (29.9) | 93.33 (38.9) | 0.300 | 90.68 (32.6) | 89.87 (35.2) | 0.866 |
| Whole Body BMD, mean (SD) | 0.056 (0.022) | 0.046 (0.025) | 0.006 | 0.052 (0.022) | 0.043 (0.026) | 0.005 |
| Lumbar BMC, mean (SD) | 2.895 (3.31) | 2.590 (3.95) | 0.588 | 2.808 (3.41) | 2.770 (3.40) | 0.939 |
| Lumbar BMD, mean (SD) | 0.011 (0.043) | 0.012 (0.043) | 0.952 | 0.006 (0.044) | 0.016 (0.043) | 0.112 |
| 1/3 Distal Radius BMC, mean (SD) | 5.526 (3.63) | 6.387 (4.46) | 0.170 | 5.255 (3.00) | 6.052 (3.46) | 0.083 |
| 1/3 Distal Radius BMD, mean (SD) | 0.033 (0.026) | 0.032 (0.027) | 0.807 | 0.032 (0.024) | 0.033 (0.027) | 0.884 |

**S5 Table. Comparison of calcaneal QUS measurements according to DXA z-score.**

|  | Whole Body BMD *z** ≤ -1.0 (n=61) | Whole Body BMD *z** > -1.0  (n=109) | p-value | Whole Body BMD *z** ≤ -2.0 (n=7) | Whole Body BMD *z** > -2.0  (n=163) | p-value | Decline in BMD z-score (n=103) | No Change/ Increase in BMD z-score (n=65) | p-value |
| --- | --- | --- | --- | --- | --- | --- | --- | --- | --- |
| ***Baseline*** | | | | | | |  |  |  |
| BUA, mean (SD) | 82.8 (13.5) | 89.5 (11.4) | 0.001 | 80.4 (13.5) | 87.3 (12.5) | 0.228 | 88.3 (12.5) | 85.0 (12.8) | 0.109 |
| SOS, mean (SD) | 1562 (19.7) | 1566 (19.3) | 0.185 | 1562 (16.0) | 1565 (19.7) | 0.679 | 1564 (19.3) | 1565 (20.2) | 0.621 |
| SI, mean (SD) | 72.5 (12.2) | 78.1 (9.99) | 0.003 | 71.0 (12.1) | 76.3 (11.1) | 0.295 | 76.8 (11.1) | 75.0 (11.4) | 0.319 |
| ***12-months**** | | | | | | |  |  |  |
|  | Whole Body BMD *z** ≤ -1.0 (n=67) | Whole Body BMD *z** > -1.0  (n=101) | p-value | Whole Body BMD *z** ≤ -2.0 (n=13) | Whole Body BMD *z** > -2.0  (n=155) | p-value | Decline in BMD (n=103) | No Change/ Increase in BMD (n=65) | p-value |
| BUA, mean (SD) | 89.9 (12.3) | 96.1 (15.2) | 0.005 | 88.7 (18.3) | 94.0 (14.0) | 0.326 | 94.0 (20.7) | 93.0 (15.5) | 0.658 |
| SOS, mean (SD) | 1567 (20.7) | 1571 (20.6) | 0.233 | 1560 (18.1) | 1570 (20.8) | 0.071 | 1569 (20.7) | 1570 (20.9) | 0.803 |
| SI, mean (SD) | 78.7 (11.9) | 84.0 (11.9) | 0.005 | 75.8 (15.5) | 82.4 (11.8) | 0.158 | 82.0 (11.3) | 81.7 (13.6) | 0.865 |

*adjusted for age, sex, race, and height-for-age z-score and whole body less head weight z-score

**
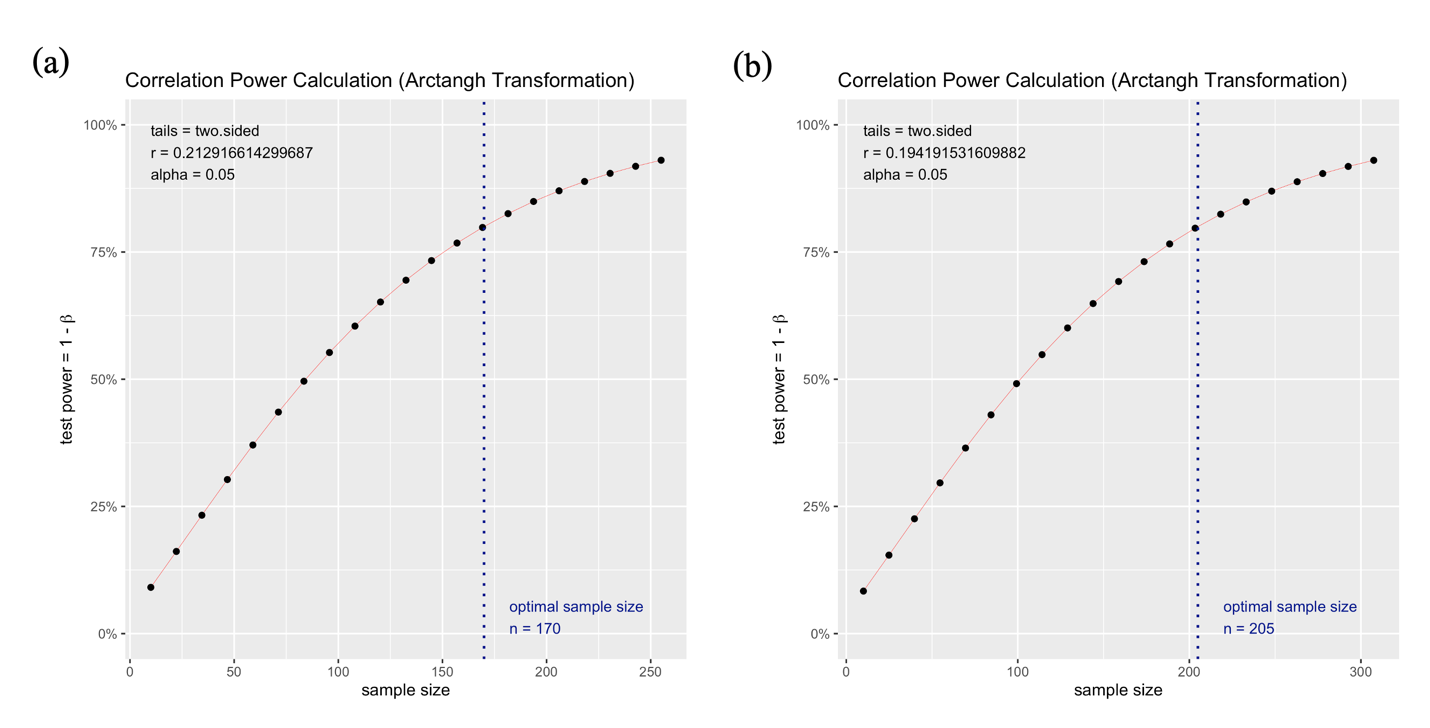
**

**S1 Fig. Power calculations for Spearman correlations.** Power calculations performed utilizing the *pwr* package in R. Null hypothesis was specified as a correlation coefficient of 0.0, power was set to 80 percent, alpha level was set to 0.05, and sample size was set to the number of participants for (a) calcaneal QUS (n = 170) and (b) radial QUS (n = 205). The estimated effect size was then calculated.

**
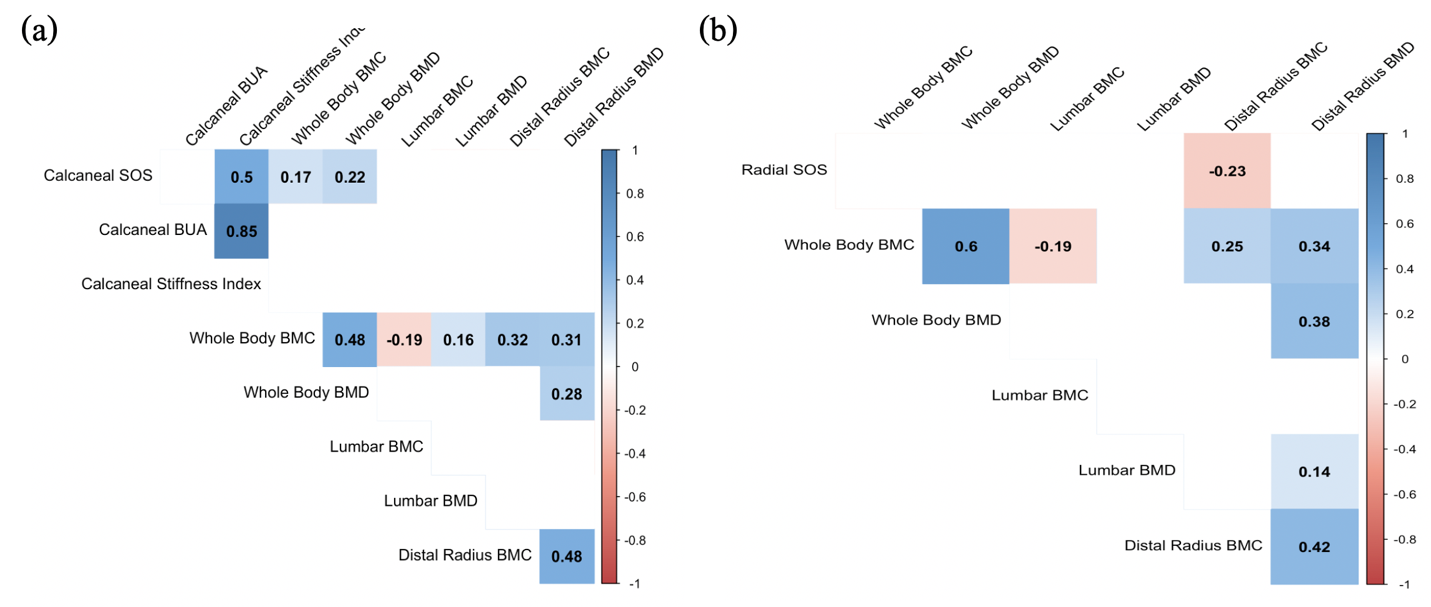
**

**S2 Fig. Pooled correlations of between QUS absolute change in measures and DXA absolute change in measures.** Correlation of the absolute changes of QUS and DXA measures between baseline and follow-up visits. (a) Results of Spearman correlation between calcaneal QUS measures and DXA (n =170). (b) Results of Spearman correlation between Mini-Omni QUS measures and DXA (n = 205). Shaded cells indicate *p* < 0.05, with darker shading indicating more significant values. Numbers included within individual cells represent Spearman rho values. BMC: bone mineral content; BMD: bone mineral density; SOS: speed of sound; DXA: dual-energy x-ray absorptiometry.


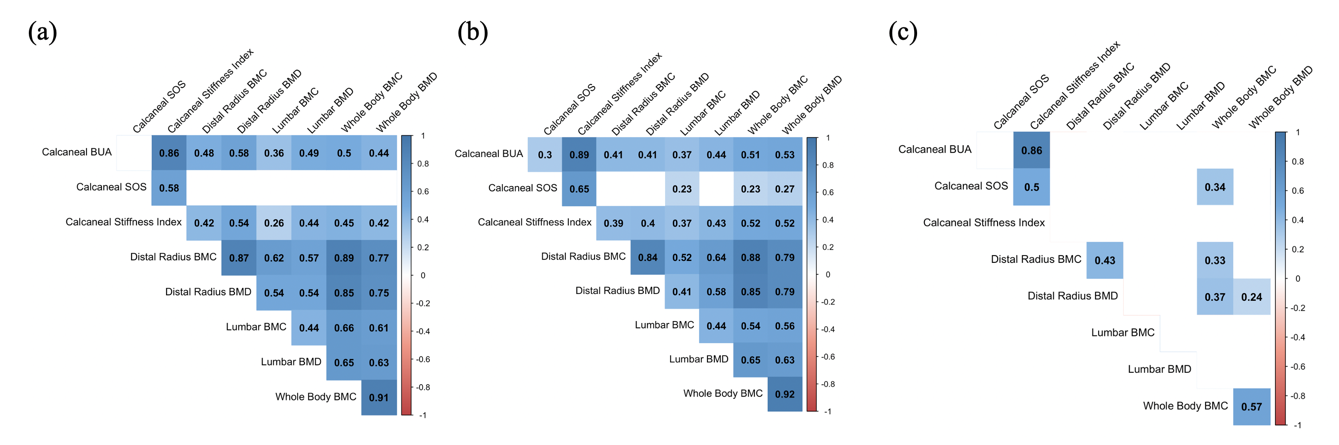


**S3 Fig. Correlations between calcaneal QUS and DXA, stratified to CLHIV.** Correlation between calcaneal QUS and DXA measures in CLHIV only. (a) Results of cross-sectional Spearman correlation between calcaneal QUS measures and DXA at Baseline (n = 80). (b) Results of cross-sectional Spearman correlation at 12-months (n=80). (c) Results of Spearman correlation between absolute change in calcaneal QUS measures from Baseline to 12-months and absolute change in DXA measures. Shaded cells indicate *p* < 0.05, with darker shading indicating more significant values. Numbers included within individual cells represent Spearman rho values. BMC: bone mineral content; BMD: bone mineral density; SOS: speed of sound; DXA: dual-energy x-ray absorptiometry.


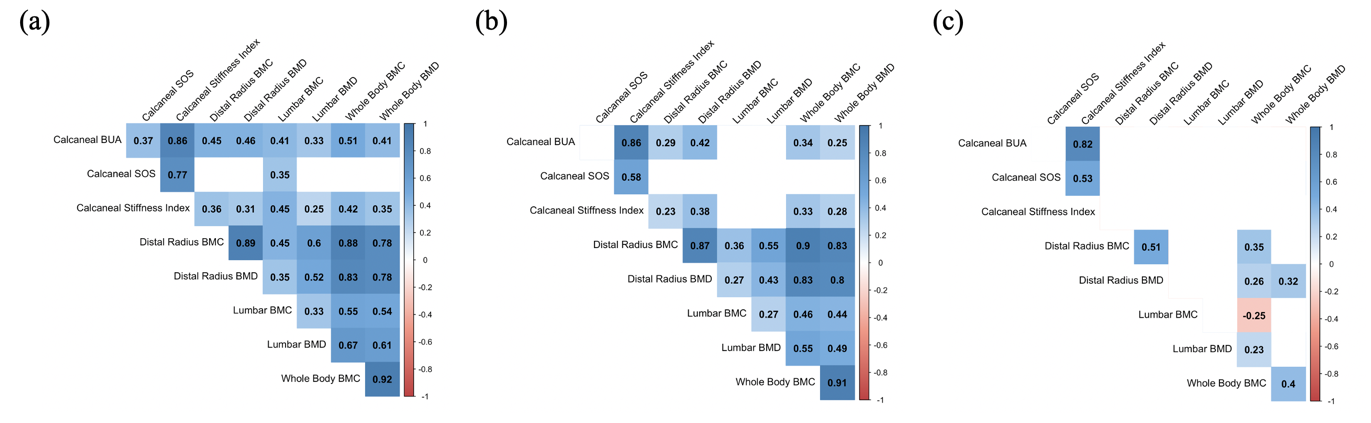


**S4 Fig. Correlations between calcaneal QUS and DXA, stratified to uninfected controls.** Correlation between calcaneal QUS and DXA measures in uninfected controls only. (a) Results of cross-sectional Spearman correlation between calcaneal QUS measures and DXA at Baseline (n = 90). (b) Results of cross-sectional Spearman correlation at 12-months (n=90). (c) Results of Spearman correlation between absolute change in calcaneal QUS measures from Baseline to 12-months and absolute change in DXA measures. Shaded cells indicate *p* < 0.05, with darker shading indicating more significant values. Numbers included within individual cells represent Spearman rho values. BMC: bone mineral content; BMD: bone mineral density; SOS: speed of sound; DXA: dual-energy x-ray absorptiometry.
