# Supplementary material for: Comparison of quantitative ultrasonography and dual X-ray absorptiometry for bone status assessment in South African children living with HIV
Source: PLoS One. 2022 Oct 17;17(10):e0276290. doi: 10.1371/journal.pone.0276290 (PMC9576091; doi:10.1371/journal.pone.0276290)
Supplement: Attachment — Submitted filename: Reviewer Response.docx [file pone.0276290.s003.docx]

The authors would like to thank the reviewers for their insightful comments, which we feel have substantially strengthened the manuscript. We have incorporated all suggested edits and have included responses to each individual reviewer’s comments below.

Reviewer #1: The manuscript conducts secondary statistical analysis to assess correlations, both cross-sectional and longitudinal, between two different methods of QUS and DXA on data generated from a previous noninferiority RCT comparing CLHIV versus controls without HIV. There are 2 longitudinal time-points: baseline, and 12-month followup. The study is based on prior science. I have some additional questions.

(a) I understand this is secondary analysis, but a sample size/power justification would assist readers to plan future trials and analysis like this. Authors may simply try to calculate the effect size they wanted to see (at the onset) to assess the differences between the groups.

To address this concern, we have calculated the effect size using the *pwr* package in R, as described in Lines 205-207. Estimated effect coefficients are included in Lines 277-280. Supplementary Figure 1 has also been added and includes a visual output of power calculations with expected effect size given our sample size and power of 80%.

(b) LOCF is really a sub-optimal method in considering missing data imputation; with several techniques available, the authors may resort to one of the credible ones. A team statistician (within the author list) is recommended, and who can be helpful in using the correct imputation method.

To address this, we have conducted an additional sensitivity analysis in which we performed multiple imputation assuming data was missing at random and have added the details to the Methods section (Lines 200-202). Results of this second sensitivity analysis were consistent with those from the last observation carried forward method, as well as the main analysis (Lines 261-265).

Reviewer #2: This reviewer has a methods concern:

1. The DXA scores were grouped: Z-score groups were z <-1.0, z < -1.5 and those who had declining z-score between visits

Please explain why the data were grouped by z-score less than -1.0 and less than -1.5. Why wasn’t the z-score of <-2.0 used, which is the internationally accepted level of what is considered to be low bone density in children. This reviewer is not convinced that there would be enough difference between the z-score less than -1.0 and less than -1.5 groups to be meaningful, particularly in this sample size.

Doubtful that this will impact the overall findings of the paper, but these groups, based on z-score, seem arbitrary and do not follow any guidelines for what is considered to be low BMD. They do not sensibly stratify the data.

We have amended the methods and results to utilize cutoffs of z <= -2, in accordance with the latest consensus guidelines, and have removed the z <= -1.5 cutoff. As there are a very small number of participants in this study with z <= -2, we have utilized the z <= -1 cutoff to capture a larger number of participants with reduced DXA scores for comparison that did not necessarily meet consensus guidelines for childhood osteoporosis. We have reflected the Methods (Lines 218-219) and Results (Lines 379-380) to reflect these changes.

2. Editorial comment: z-scores should be expressed as a decimal (e.g. -1.0). In this paper, z-scores are expressed as -1.

We have edited the paper to include decimals for z-scores.
